# Supported MXene/GO Composite Membranes with Suppressed Swelling for Metal Ion Sieving

**DOI:** 10.3390/membranes11080621

**Published:** 2021-08-13

**Authors:** Zongjie Yin, Zong Lu, Yanyan Xu, Yonghong Zhang, Liliang He, Peishan Li, Lei Xiong, Li Ding, Yanying Wei, Haihui Wang

**Affiliations:** 1 School of Chemistry and Chemical Engineering, South China University of Technology, Guangzhou 510640, China; zongjieyin@126.com (Z.Y.); zongluscut@163.com (Z.L.); dingli@scut.edu.cn (L.D.); 2 Guangzhou Special Pressure Equipment Inspection and Research Institute, Guangzhou 510000, China; lylxyy66@163.com (Y.X.); zhangyonghong81@163.com (Y.Z.); helilianga@163.com (L.H.); san_12dy@163.com (P.L.); xionglei.888@163.com (L.X.); 3 Beijing Key Laboratory for Membrane Materials and Engineering, Department of Chemical Engineering, Tsinghua University, Beijing 100084, China

**Keywords:** membrane separation, two-dimensional membrane, MXene, GO, ion rejection, swelling, supported membrane

## Abstract

Novel two-dimensional (2D) membranes have been utilized in water purification or seawater desalination due to their highly designable structure. However, they usually suffer from swelling problems when immersed in solution, which limits their further applications. In this study, 2D cross-linked MXene/GO composite membranes supported on porous polyamide substrates are proposed to improve the antiswelling property and enhance the ion-sieving performance. Transition-metal carbide (MXene) nanosheets were intercalated into GO nanosheets, where the carboxyl groups of GO combined the neighboring hydroxyl terminal groups of MXene with the formation of -COO- bonds between GO and MXene nanosheets via the cross-linking reaction (−OH + −COOH = −COO− + H_2_O) after heat treatment. The permeation rates of the metal ions (Li^+^, Na^+^, K^+^, Al^3+^) through the cross-linked MXene/GO composite membrane were 7–40 times lower than those through the pristine MXene/GO membrane. In addition, the cross-linked MXene/GO composite membrane showed excellent Na^+^ rejection performance (99.3%), which was significantly higher than that through pristine MXene/GO composite membranes (80.8%), showing improved ion exclusion performance. Such a strategy represents a new avenue to develop 2D material-derived high-performance membranes for water purification.

## 1. Introduction

Membrane-based separation technology has played an increasingly important role in water purification because of its cost-effectiveness, energy efficiency, and easy operation [1,2,3,4,5]. In particular, two-dimensional (2D) nanosheet membranes have attracted intense attention due to their excellent mechanical properties and adjustable molecule/ion sieving ability [6,7,8,9,10,11]. In recent years, 2D membranes have been widely studied in molecular sieving, including gas, metal ions, solvent, dye, etc. [12,13].

As one kind of crucial 2D nanomaterial, graphene oxide (GO), has great potential for separation application processes [14]. Due to their superior ion selectivity, good mechanical strength, versatile chemical modification, and antifouling potential, GO membranes are promising for water purification [6,15,16,17,18,19,20,21]. Moreover, MXene, an emerging family of 2D transition metal carbides, and nitrides, breaks new ground for membrane separation [7,22,23,24,25,26,27], whose abundant surface functional groups provide more possibilities for structural design. Ti_3_C_2_T_x_, as the most studied MXene (its detailed structure can be found in Figure S1 of Supporting Information), has been used to assemble 2D lamellar membranes for different separation applications [7,28,29,30], especially in water treatment [22,28,30]. 

However, there is one big challenge for 2D lamellar membranes applied in the field of ion rejection. When immersed in water or salt solutions, most 2D membranes tend to absorb water molecules, leading to increased d-spacing and thus decreased stability and ion rejection performance, which is known as the notorious swelling problem [31,32,33,34,35,36]. Hence, suppressing the swelling is of great importance to improve the ion sieving ability of 2D membranes. In the last few years, many strategies have been used to enhance the water stability of GO membranes, and the most common method is to incorporate other nanomaterials, such as graphene, graphitic carbon nitride, or carbon nanotubes into the GO membranes [37,38,39]. Great efforts have been made to solve the swelling problems of 2D membranes, mainly by fixing the interlayer spacing using cross-linking agents [40,41,42]. However, most cross-linking processes are always too complicated to scale up. Therefore, new ways are needed to improve the antiswelling properties of 2D membranes.

**Figure 1:**
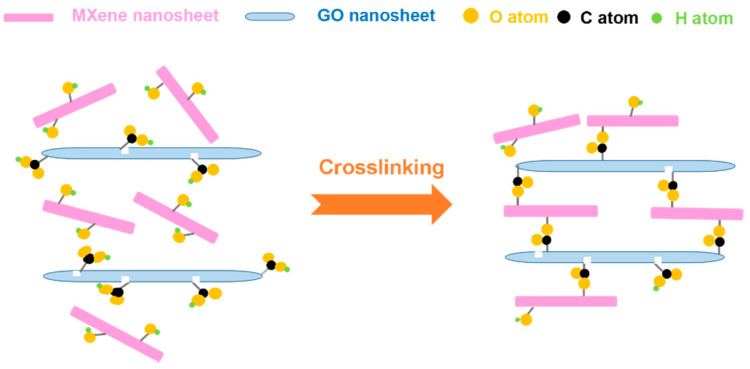
Cross-linking process between the neighboring GO nanosheets and MXene nanosheets in the MXene/GO composite membranes.

Here, we propose MXene (Ti_3_C_2_T_x_)-GO composite membranes for ion sieving. It was reported that Ti_3_C_2_T_x_ could easily enter the graphene sheet layer when Ti_3_C_2_T_x_ was combined with graphene [43]. Furthermore, there are abundant surface functional groups (O, -OH, and -F) on the nanosheet surface of MXene. The carboxyl groups on the GO nanosheets [21] are expected to react with the hydroxyl groups of MXene nanosheets to form -COO- in the interlayer sub-nanochannels. As a result, the swelling behavior of the MXene-GO membranes would be hindered, which is beneficial for the MXene-GO membranes to block metal ions. As shown in Figure 1, herein, the cross-linked MXene/GO composite membranes have been successfully prepared via −OH + −COOH = −COO− + H_2_O between the neighboring GO nanosheets and MXene nanosheets after heat treatment followed by vacuum filtration of the mixed MXene and GO solutions. The cross-linked MXene/GO composite membranes exhibit a significantly improved ion sieving performance with suppressed swelling compared to the pristine MXene/GO composite membranes before cross-linking.

## 2. Materials and Methods

### 2.1. Materials

The preparation of the MXene nanosheet solution, the raw materials used in this process and the calculation of its concentration are described in our previous work [44]. The GO nanosheets were purchased from Nanjing Jicang Nano Technology Co. Ltd., Nanjing, China. The porous polyamide was obtained from Jinteng Experimental Equipment Co., LTD (Tianjin, China) with a diameter of 0.45 mm and pore size of 0.22 μm.

### 2.2. Preparation of the Pristine MXene/GO Composite Membranes

A certain amount of MXene solution (1 mg mL^−1^) was mixed with 25% GO solution (1 mg mL^−1^) and stirred for 30 min to obtain a homogenous mixed MXene/GO solution. Further studies on composite membranes with various ratios are still ongoing. The pristine MXene/GO composite membranes were prepared by vacuum-assisted filtration of the MXene/GO solution on the porous polyamide substrate. Then the membranes were dried in a vacuum dryer at room temperature (25 °C) for 12 h. During membrane preparation, the length of time of vacuum-assisted filtration will affect the tightness between the nanosheets, and thus affect the ion rejection performance of the membranes. Therefore, we strictly ensured a uniform vacuum-assisted filtration time of each membrane to eliminate this factor.

### 2.3. Preparation of the Cross-Linked MXene/GO Composite Membranes

After room temperature drying, the membrane was transferred to a drying oven for cross-linking treatment, where the oven temperature was controlled exactly at 170 °C with feedback mode via thermocouple. Then the cross-linked MXene/GO composite membranes were obtained after heat treatment at 170 °C for 12 h in a vacuum drying oven for cross-linking followed by cooling down to room temperature. The membrane thicknesses before and after heat treatment were 447 nm and 317 nm, respectively (detailed calculation is shown in the Supplementary Note S1).

### 2.4. Ion Permeation

**Figure 2:**
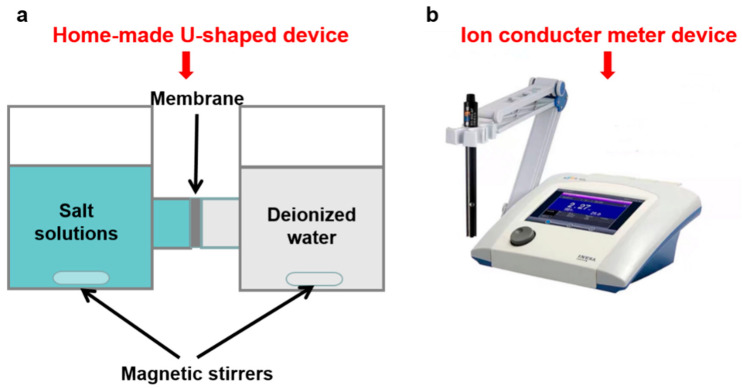
(**a**) The home-made U-shaped device for ion permeation test. (**b**) Photo of the ion conductor meter device.

The measurement of the ion permeation of the MXene/GO composite membranes was carried out via a homemade U-shaped device (Figure 2a). Before measurement, the membranes were sealed in the middle of the device, and the feed cabin and permeation cabin were filled with salt solution (0.2 M) and DI water, respectively. In addition, the solution in both cabins was magnetically stirred to avoid the concentration polarization effect near the membrane. The ion permeation rates were calculated via the ionic conductivity detected by the ion conductivity meter (DDSJ-319L, Shanghai Leici Instrument Factory, Shanghai, China) (Figure 2b). Calculation details of the ion rejection are described in our previous work [44].

### 2.5. Characterizations

The atomic force microscopy (AFM) images were obtained using a Bruker Dimension Icon scanning probe microscope (SPM) in PeakForce tapping mode. The scanning electron microscopy (SEM) images were obtained from the Hitachi SU8220 device (Ibaraki, Japan). The water contact angle was measured using an automatic contact angle measuring instrument (Biolin, Attension Theta, Gothenburg, Sweden). The X-ray photoelectron spectroscopy (XPS) analysis was carried out using a theta probe spectrometer (Thermo Fisher, Brno, Czech Republic) with monochromatic Al-Kα radiation (1486.6 eV). Raman spectroscopy was performed on a Renishaw inVia Reflex Raman microscope (London, England) with 633 nm laser excitation. The FTIR characterization was carried out using a Thermofisher IS50 spectrometer (Brno, Czech Republic) in attenuated total reflection (ATR) mode in the wavenumber range of 400–4000 cm^−1^. The X-ray diffraction (XRD) analysis was performed using Rigaku Smart Lab X-Ray Diffractometer (Japan) with filtered Cu-Kα radiation (40 kV and 40 mA, λ = 0.154 nm).

## 3. Results and Discussion

### 3.1. Characterization of the MXene Nanosheets and GO Nanosheets

**Figure 3:**
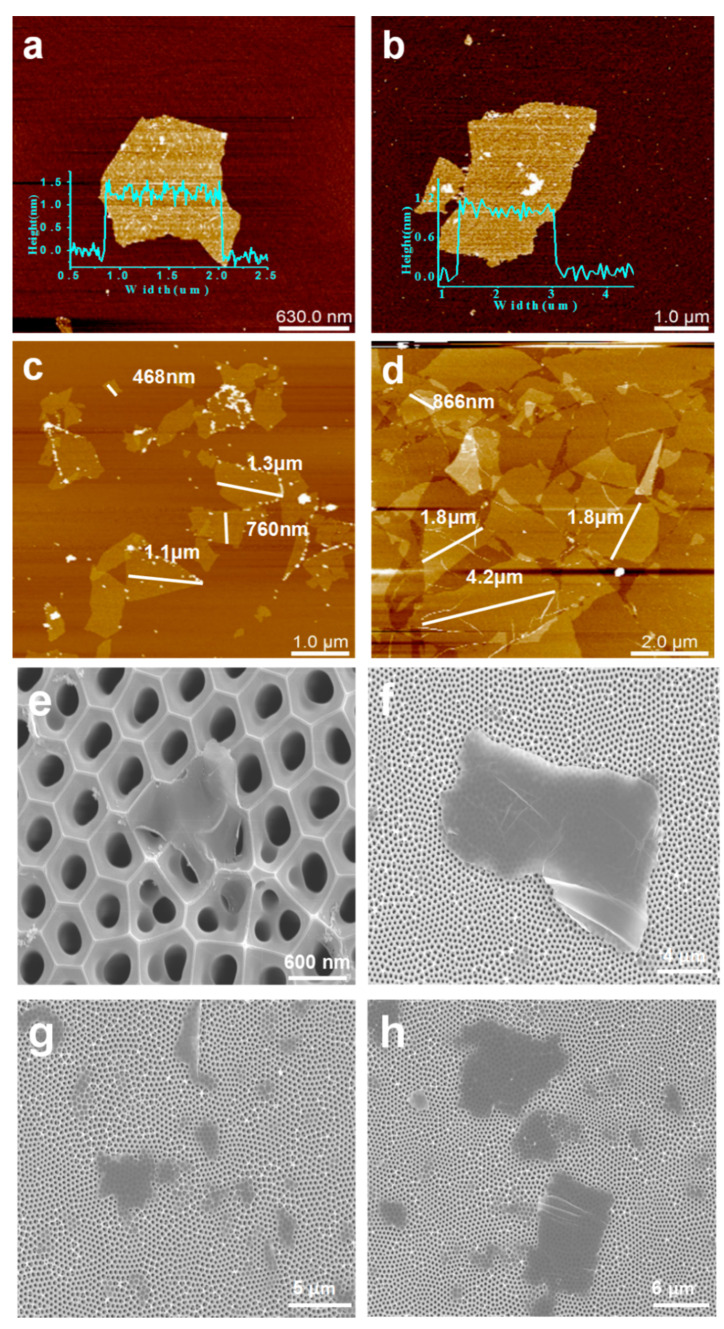
AFM images of (**a**,**c**) the MXene nanosheets and (**b**,**d**) the GO nanosheets. SEM images of (**e**,**g**) the MXene nanosheets and (**f**,**h**) the GO nanosheets.

The AFM images (Figure 3a–d) and the SEM images (Figure 3e–h) of the MXene and GO nanosheets indicate that the MXene nanosheets exhibited an average thickness of ~1.3 nm with a lateral size in the range of several hundreds of nanometers to a few microns, while the average thickness of the GO nanosheets was ~1.2 nm with lateral dimensions ranging from a few microns to a dozen microns.

**Figure 4:**
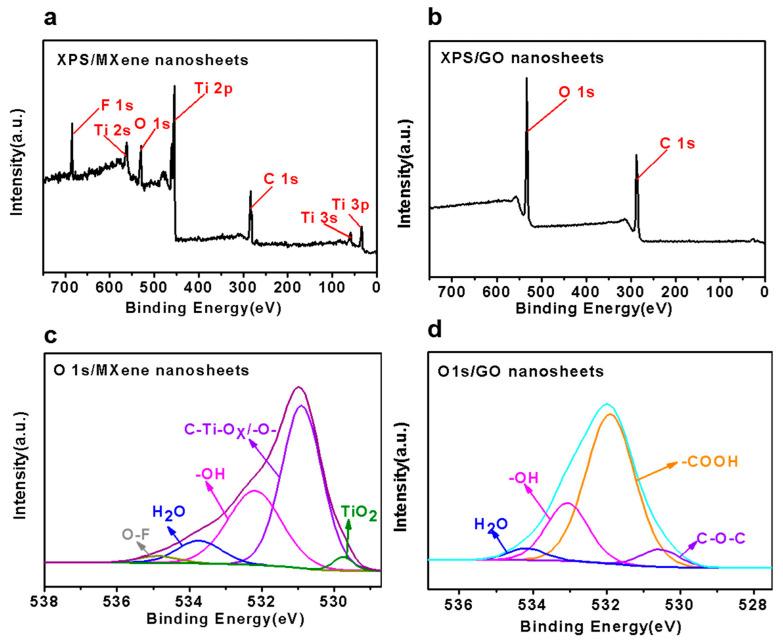
The XPS results of (**a**) MXene nanosheets and (**b**) GO nanosheets. Component peak fitting of the XPS spectra for O 1s of (**c**) MXene nanosheets and (**d**) GO nanosheets.

To further characterize the materials, XPS analysis of the MXene and GO nanosheets was conducted. The XPS spectra shown in Figure 4a,b reveal that the MXene nanosheet is made up of C, O, F and Ti, while the GO nanosheet contains C and O. Furthermore, it was identified from Figure 4c,d that MXene was rich in terminating functional groups, while GO was rich in groups of −COOH, −OH, and C−O−C. In particular, the hydroxyl groups on the MXene nanosheets and the carboxyl groups on the GO nanosheets made cross-linking possible between the neighboring GO nanosheets and MXene nanosheets in this composite membrane.

### 3.2. Characterization of the MXene/GO Composite Membranes

**Figure 5:**
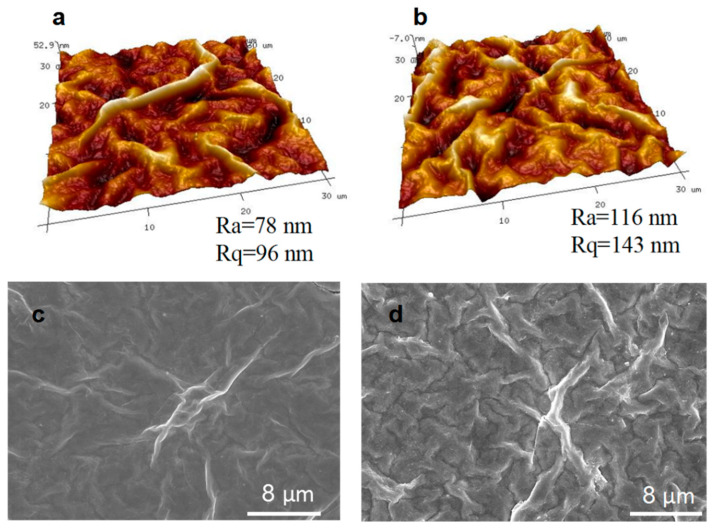
AFM and SEM images of the pristine MXene/GO composite membrane and cross-linked MXene/GO composite membranes. AFM images of (**a**) pristine MXene/GO composite membranes and (**b**) cross-linked MXene/GO composite membranes. SEM images of (**c**) pristine MXene/GO composite membranes surface and (**d**) cross-linked MXene/GO composite membranes surface.

The AFM (Figure 5a,b) and SEM (Figure 5c,d) images show that the surface of the cross-linked MXene/GO composite membranes became significantly rougher after thermal treatment. The roughness parameters of Rq (root-mean-square roughness) and Ra (arithmetic average roughness) increased from 96 and 78 nm to 143 and 116 nm, respectively. Besides that, the water contact angle (Figure S2) of the cross-linked MXene/GO composite membranes slightly increased compared to the pristine MXene/GO composite membranes, due to the esterification during cross-linking between the neighboring MXene nanosheets and GO nanosheets, as well as the dehydration and decrease of oxygen-containing functional groups in the crosslinking process. The increased number of hydrophobic channels in the cross-linked MXene/GO composite membrane were also more conducive for the blocking of hydrated ions.

**Figure 6:**
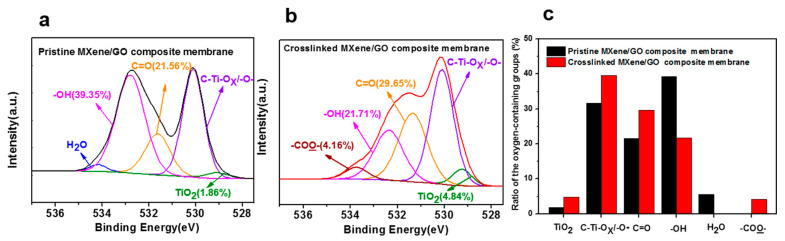
Component peak fitting of the XPS spectra for O 1s of (**a**) pristine MXene/GO composite membrane and (**b**) cross-linked MXene/GO composite membrane; (**c**) the ratio of the oxygen-containing groups in the pristine MXene/GO composite membrane and cross-linked MXene/GO composite membrane.

**Figure 7:**
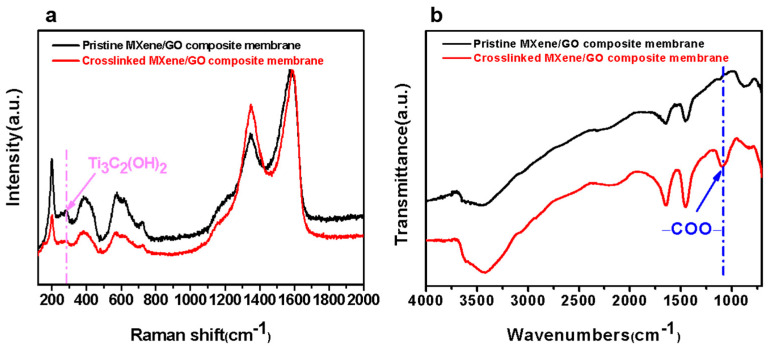
(**a**) Raman spectra of the pristine MXene/GO composite membrane and cross-linked MXene/GO composite membrane. (**b**) FTIR spectra of the pristine MXene/GO composite membrane and cross-linked MXene/GO composite membrane.

To further prove the formation of the −COO− bonds, a comparison of the XPS analysis results of the pristine MXene/GO composite membrane and the cross-linked MXene/GO composite membrane was performed. The O 1s region of the pristine MXene/GO composite membrane shown in Figure 6 exhibited that the fraction of TiO_2_ of the pristine MXene/GO composite membranes increased only slightly after thermal treatment, indicating that the composite membrane was barely oxidized, showing excellent stability. In addition, the fraction of −OH of the pristine MXene/GO composite membranes was 39.35%. After the thermal cross-linking process, the −OH fraction decreased to 21.71%. Although it was not enough to prove that the amount of -OH had definitely decreased because the fractions mentioned here are relative, considering that the amount of stable C=O does not change during thermal treatment, the ratio of −OH to C=O fraction decreases from 1.82 to 0.73, indicating that the -OH content of the composite membrane indeed decreases after thermal treatment. The Raman results (Figure 7a) also demonstrated this change. The peak at 284 cm^−1^ was assigned to the Eg mode of Ti_3_C_2_(OH)_2_ [44] and compared to the pristine MXene/GO composite membrane, this peak of the cross-linked MXene/GO composite membrane decreased obviously, showing the consumption of -OH. More importantly, the −COO− peak appeared with a fraction of 4.16% in the cross-linked MXene/GO composite membrane, as shown in Figure 6b. Furthermore, compared to the FTIR result of the pristine MXene/GO composite membrane shown in Figure 7b, an obvious peak at the wavenumber of ~1091 cm^−1^ can be found in that of the cross-linked MXene/GO composite membrane, which can be attributed to the stretching vibrations of the −COO− bond, further indicating the formation of −COO− bonds between the MXene nanosheets and GO nanosheets in the cross-linked MXene/GO composite membrane, which further confirms the process of esterification.

**Figure 8:**
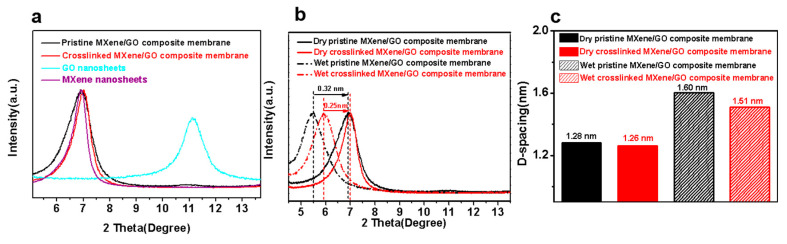
(**a**) XRD patterns of the MXene/GO composite membrane, GO nanosheets and MXene nanosheets. (**b**) XRD patterns of the pristine and cross-linked MXene/GO composite membrane in dry and wet state. (**c**) The d-spacing of the pristine and cross-linked MXene/GO composite membranes in dry and wet state.

Furthermore, the XRD analysis has also proved the cross-linking reaction within the MXene/GO composite membrane. As shown in Figure 8a, the peak of the GO nanosheets was almost absent in the XRD patterns of the MXene/GO composite membranes because of their low content compared to the MXene nanosheets. Therefore, only the peak belonging to the MXene nanosheets was chosen as the representative peak of the MXene/GO composite membrane for further analysis. As shown in Figure 8b and c, the d-spacing of the MXene/GO composite membrane calculated from Bragg’s equation slightly decreased from 1.28 to 1.26 nm after cross-linking in dry state. The d-spacing of the pristine MXene/GO composite membrane expanded to 1.60 nm after immersing in water for 20 h, due to swelling, while the d-spacing of the cross-linked MXene/GO composite membrane could be maintained at 1.51 nm even in a wet state, which can be attributed to the swelling being suppressed, due to the cross-linking reaction via −OH + −COOH = −COO− + H_2_O. Moreover, the interlayer spacing change of the membrane with time of both the pristine and the cross-linked MXene/GO composite membranes is shown in Figure S3 in Supporting Information. Both of the d-spacings increased with the length of time of membrane immersion in water, due to avoidable swelling of the lamellar membrane. The d-spacing could almost reach a steady value when the immersion time was longer than 2 h. However, it should be noted that the interlayer spacing of the cross-linked MXene/GO composite membrane was much smaller than that of the pristine one due to the obviously suppressed swelling.

### 3.3. Ion Exclusion Performance of the MXene/GO Composite Membranes

**Figure 9:**
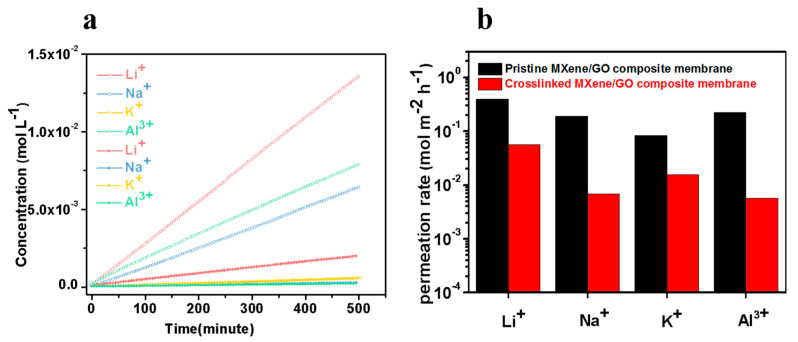
(**a**) The scatter diagram of permeation rates of Li^+^, Na^+^, K^+^ and Al^3+^ through the pristine MXene/GO composite membrane and cross−linked MXene/GO composite membrane. The hollow sphere curve represents the ion penetration rate of the pristine MXene/GO composite membrane, while the solid one represents the ion penetration rate of the cross−linked MXene/GO composite membrane. (**b**) The histogram of permeation rates of Li^+^, Na^+^, K^+^ and Al^3+^ through the pristine MXene/GO composite membrane and cross-linked MXene/GO composite membrane.

The permeation rates of four kinds of metal ions through the pristine MXene/GO composite membranes and cross-linked MXene/GO composite membranes were measured. As shown in Figure 9, the pristine MXene/GO composite membranes offered permeation rates of Li^+^ (hydrated diameter of 7.64 Å), Na^+^ (hydrated diameter of 7.16 Å), K^+^ (hydrated diameter of 6.62 Å), Al^3+^ (hydrated diameter of 9.50 Å) [28] of 0.395, 0.191, 0.0833 and 0.226 mol h^−1^ m^−2^, respectively. On the basis of the previous XRD results shown in Figure 8c, the effective nanochannel height for mass transport channel between neighboring nanosheets can be calculated from the d-spacing (deduced by Bragg equation) by subtracting the thickness of nanosheet, where both the monolayer MXene and the few layered GO nanosheets are ~1 nm [44]. Therefore, the effective distances for mass transport in the pristine MXene/GO composite membrane and the cross-linked MXene/GO composite membrane immersed in solution are 6.0 Å and 5.1 Å, respectively. It was found that the swollen pristine MXene/GO composite membrane with the effective nanochannel height of 6.0 Å had no obvious exclusion performance for the metal ions due to the partial dehydration of the dehydrated ions [34].

In contrast, the cross-linked MXene/GO composite membranes exhibited significantly reduced ion permeation rates, where the permeation rates of Li^+^, Na^+^, K^+^, Al^3+^ were (5.71, 0.688, 1.58 and 0.57) × 10^−2^ mol h^−1^ m^−2^, respectively, which were about one to two orders of magnitudes lower than that through the pristine MXene/GO composite membranes. In other words, the ion rejection performance of the cross-linked MXene/GO composite membrane was greatly improved, due to the formation of −COO− bonds, which is beneficial for obtaining relatively stable sub-nanochannels in solutions after cross-linking. Moreover, it should be noted that all of the hydrated metal ions would partially dehydrate when entering the nanochannels of the membrane. Therefore, the transport behavior of the hydration ions through the membrane with a narrower interlayer spacing is determined by the energy barrier associated with dehydration. That is why the permeation rates of K^+^, Na^+^, Li^+^ and Al^3+^ did not show this tendency with the order of their hydration diameters, which was also in accordance with our previous work [44]. Herein, the permeation behavior of an ion was mainly determined by the hydration size, so, the large, hydrated diameter (9.50 Å) of Al^3+^ resulted in a low permeation rate. Al^3+^ needed to overcome a large dehydration energy barrier through the nanochannels of the cross-linked MXene/GO composite membrane, and more bound water molecules needed to be removed, leading to the large drop of the permeation rate of Al^3+^. On the other hand, the higher positive charge of Al^3+^ would promote its transport through the membrane due to the Donnan effect, because the membrane surface is negatively charged, which is not beneficial for ion rejection. From this point of view, the 3+ charge on the Al ion barely influenced its large drop on permeation rate, but the relatively higher dehydration energy barrier of Al^3+^ worked. It can be seen from Table S1 in Supporting Information that the cross-linked MXene/GO composite membrane prepared in this work had good ion rejection performance. Additionally, as is known, the ion permeation rate increases with the decreasing membrane thickness due to an unavoidable defect. However, the ion permeation rate even decreased through the thinner MXene/GO composite membrane after cross-linking, which can be attributed to the better membrane structure with suppressed swelling rather than the influence of thickness change.

## 4. Conclusions

We propose a type of cross-linked MXene/GO composite membrane with enhanced ion exclusion performance. The hydroxyl groups on the MXene nanosheets and the carboxyl groups on the GO nanosheets within the composite membrane tend to react and form –COO– bonds to connect neighboring nanosheets tightly even in water or salt solutions, showing obviously suppressed swelling, which the XRD results can intuitively confirm. The XPS, FTIR and Raman characterizations confirm the decrease of hydroxyl groups and the formation of −COO− bonds, demonstrating the occurrence of esterification during cross-linking of the MXene/GO composite membrane. Compared to that of the pristine membranes, the permeation rate of the ions (K^+^, Na^+^, Li^+^, Al^3+^) through the cross-linked MXene/GO composite membranes was reduced by at least one order of magnitude, i.e., the ion sieving performance of the membranes was improved 7–40 times after cross-linking treatment. Therefore, such cross-linked MXene/GO composite membranes represent a new avenue to develop 2D material-derived high-performance membranes for water purification.

## Data Availability

Not applicable.

## Supplementary Materials

The following are available online at https://www.mdpi.com/article/10.3390/membranes11080621/s1, Figure S1: The schematic illustration of the structure of MXene (Ti3C2Tx); Figure S2: The water contact angle of five randomly selected points on the pristine MXene/GO composite membrane and cross-linked MXene/GO composite membrane; Figure S3: (a) XRD patterns of the pristine MXene/GO composite membranes after immersing in water with different time. (b) XRD patterns of the cross-linked MXene/GO composite membranes after immersing in water with different time. (c) Trace of the d-spacing of the pristine and cross-linked MXene/GO composite membranes with different immersing time; Table S1. Comparison of desalination performance of various lamellar membranes from literatures; Note S1: The calculation method of the thickness of the pristine MXene/GO composite membrane.
