# Supplementary material for: Supported MXene/GO Composite Membranes with Suppressed Swelling for Metal Ion Sieving
Source: Membranes (Basel). 2021 Aug 13;11(8):621. doi: 10.3390/membranes11080621 (PMC8401878; doi:10.3390/membranes11080621)
Supplement: Supplementary file 1 [file membranes-11-00621-s001.zip › membranes-1292910-supplementary.pdf]

membranes

Supplementary Materials: Supported MXene/GO Composite Membranes with Suppressed Swelling for Metal Ion Sieving

Zongjie Yin [1,2,†], Zong Lu [1,†], Yanyan Xu [2], Yonghong Zhang [2], Liliang He [2], Peishan Li [2], Lei Xiong [2], Li Ding [1], Yanying Wei [1,*] and Haihui Wang [3,*]

[1] School of Chemistry and Chemical Engineering, South China University of Technology, 510640 Guangzhou, China; zongjieyin@126.com (Z.Y.); zongluscut@163.com (Z.L.); dingli@scut.edu.cn (L.D.)

[2] Guangzhou Special Pressure Equipment Inspection and Research Institute, Guangzhou, 510000, China; lylxyy66@163.com (Y.X.); zhangyonghong81@163.com (Y.Z.); helilianga@163.com (L.H.); san_12dy@163.com (P.L.); xionglei.888@163.com (L.X.)

[3] Beijing Key Laboratory for Membrane Materials and Engineering, Department of Chemical Engineering, Tsinghua University, 100084 Beijing, China

* Correspondence: ceyywei@scut.edu.cn (Y.W.); cehhwang@tsinghua.edu.cn (H.W.)

† These authors contribute equally to this work.

1. Supplementary Figures

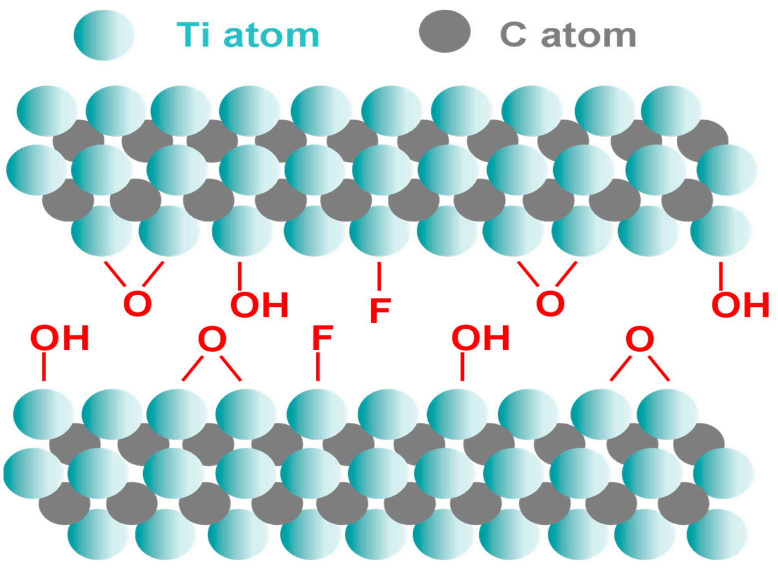

Figure S1. The schematic illustration of the structure of MXene ($Ti_3C_2T_x$).

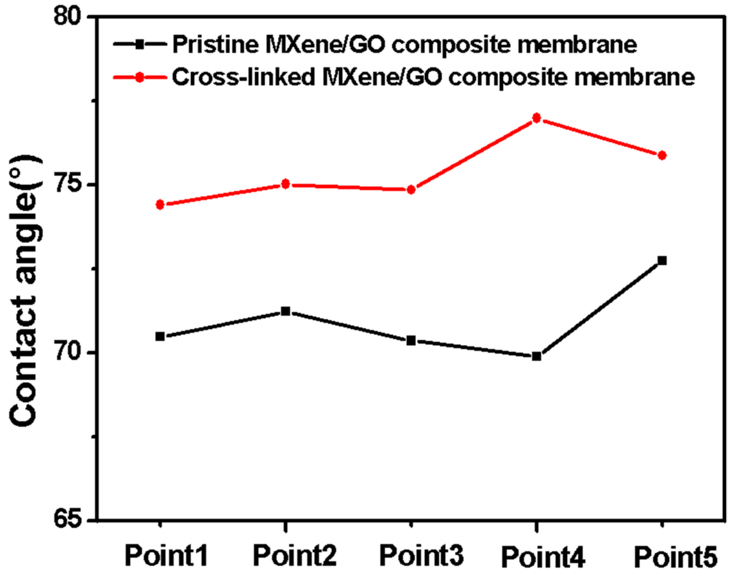

Figure S2. The water contact angle of five randomly selected points on the pristine MXene/GO composite membrane and cross-linked MXene/GO composite membrane.

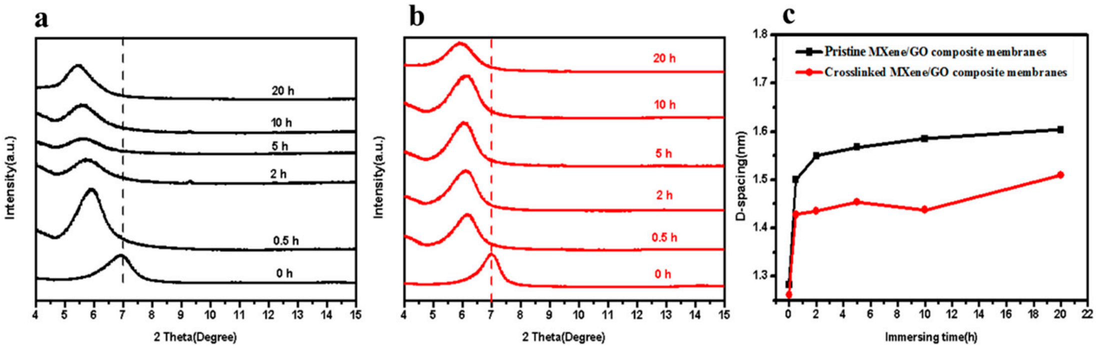

Figure S3. (a) XRD patterns of the pristine MXene/GO composite membranes after immersing in water with different time. (b) XRD patterns of the cross-linked MXene/GO composite membranes after immersing in water with different time. (c) Trace of the d-spacing of the pristine and cross-linked MXene/GO composite membranes with different immersing time.

2. Supplementary Table

Table S1. Comparison of desalination performance of various lamellar membranes from literatures.

Membrane	Feed side	NaCl rejection (%)	Ref.
PIP	2.5mM	34.91	[1]
PIP-GO		31.58	
TMPyP/GO	0.034 M NaCl	~25	[2]
15-layered GO membrane	0.1mM	59	[3]
	1mM	29	
GO	0.02 M NaCl	40	[4]
PEI modified GO/PAA/PVA/GA	0.034 M NaCl	43.2	[5]
Al^{3+}-intercalated MXMs	0.1 M NaCl	89.5	[6]
PMM	0.1 M NaCl	55	[7]
SCMM-80		97	
SCMM-120		98	
The pristine MXene/GO composite membrane	0.2M NaCl	80.8	This work
The cross-linked MXene/GO composite membrane		99.3	

3. Supplementary Notes

Note S1: The calculation method of the thickness of the pristine MXene/GO composite membrane.

The thickness of the pristine MXene/GO composite membrane was calculated according to the loading amount combined with the surface area of the membrane. In this work, the diameter of all membranes prepared are uniformly 20 mm. The thickness of the pristine MXene/GO composite membrane (d) (μm) can be estimated by the formula as follows:

$$d = m \times 0.894 \quad (1)$$

where m is the loading amount of 2D nanosheets (MXene and GO) (mg), 0.894 is an empirical coefficient in our series of work of lamellar membranes.

References

1. Hu, R.; He, Y.; Zhang, C.; Zhang, R.; Li, J.; Zhu, H. Graphene oxide embedded polyamide nanofiltration membranes for selective ion separation. J Mater Chem A. 2017, 25632-25640.
2. Barsoum, M. W. The $M_{N+1}AX_N$ phases: A new class of solids: Thermodynamically Stable Nanolaminates. Prog. Solid State Chem. 2000, 28, 201-281.
3. Hu, M.; Mi, B. Enabling graphene oxide nanosheets as water separation membranes. Environ. Sci. Technol. 2013, 47, 3715-3723..
4. Hope, M. A.; Forse, A. C.; Griffith, K. J.; Lukatskaya, M. R.; Ghidiu, M.; Gogotsi, Y.; Grey, C. P. NMR Reveals the surface functionalisation of Ti_3C_2 MXene. Phys. Chem. Chem. Phys. 2016, 18, 5099-5102.
5. Ding, L.; Wei, Y.; Li, L.; Zhang, T.; Wang, H.; Xue, J.; Ding, L.; Wang, S.; Caro, J.; Gogotsi, Y. MXene molecular sieving membranes for highly efficient gas separation. NAT Commun. 2018, 9(1), 435-676.
6. Ding, L.; Li, L.; Liu, Y.; Wu Y; Lu, Z.; Deng, J.; Wei, Y.; Caro, J.; Wang, H. Effective ion sieving with $Ti_3C_2T_x$ MXene membranes for production of drinking water from seawater. NAT Sustain. 2020, 3(4), 296-302.
7. Lu, Z.; Wei, Y.; Deng, J.; Ding, L.; Li, Z.; Wang, H. Self-crosslinked MXene ($Ti_3C_2T_x$) membranes with good antiswelling property for monovalent metal ion exclusion. ACS Nano. 2019, 13, 10535-10544.
